# Unresectable localized neuroblastoma: improved survival after primary chemotherapy including carboplatin-etoposide. Neuroblastoma Study Group of the SociÃ©tÃ© FranÃ§aise d'Oncologie PÃ©diatrique (SFOP).

**DOI:** 10.1038/bjc.1998.384

**Published:** 1998-06

**Authors:** H. Rubie, J. Michon, D. Plantaz, M. C. Peyroulet, C. Coze, D. Frappaz, P. Chastagner, M. C. Baranzelli, F. MÃ©chinaud, P. Boutard, P. Lutz, Y. Perel, G. Leverger, L. de Lumley, F. Millot, J. L. StÃ©phan, G. Margueritte, O. Hartmann

**Affiliations:** UnitÃ© d' HÃ©mato-Oncologie PÃ©diatrique, Service de MÃ©decine Infantile B, CHU Purpan, Toulouse, France.

## Abstract

Neuroblastomas (NBs) were assessed according to INSS recommendations including MIBG scan and extensive bone marrow staging to eliminate metastatic spread. Patients with unresectable tumour received primary chemotherapy including two courses of carboplatin-etoposide (CE) and two of vincristine-cyclophosphamide-doxorubicin (CAdO). Post-operative treatment was to be given only in children over 1 year of age at diagnosis who had residual disease or lymph node (LN) involvement. Between 1990 and 1994, 130 consecutive children were registered. In comparison with resectable primaries, these tumours were more commonly abdominal, larger and associated with N-myc amplification (NMA). Complete, very good and partial response (CR, VGPR, PR) to CE were, respectively, 1%, 7% and 44%, overall response rate (RR) to two courses of CE and two courses of CAdO was 71%, and the tumour could be removed in all but four of the children. The toxicity was manageable. The 5-year overall survival (OS) and event-free survival (EFS) were, respectively, 88% and 78% with a median follow-up of 38 months. In multivariate analysis, only NMA and LN involvement adversely influenced the outcome, particularly NMA. Children with unresectable NBs and no NMA fared as well as children with resectable ones as OS were, respectively, 95% and 99% and EFS 89% and 91%. Our data show encouraging results in localized but unresectable NBs as 90% of children may be considered as definitely cured, especially those without NMA.


British Journal of Cancer (1998) 77(12), 2310–2317
© 1998 Cancer Research Campaign

### Unresectable localized neuroblastoma: improved survival after primary chemotherapy including carboplatin–etoposide

H Rubie, J Michon, D Plantaz, MC Peyroulet, C Coze, D Frappaz, P Chastagner, MC Baranzelli, F Méchinaud, P Boutard, P Lutz, Y Perel, G Leverger, L de Lumley, F Millot, JL Stéphan, G Marguerite and O Hartmann for the Neuroblastoma Study Group of the Société Française d'Oncologie Pédiatrique (SFOP)

**Summary** Neuroblastomas (NBs) were assessed according to INSS recommendations including MIBG scan and extensive bone marrow staging to eliminate metastatic spread. Patients with unresectable tumour received primary chemotherapy including two courses of carboplatin–etoposide (CE) and two of vincristine–cyclophosphamide–doxorubicin (CAdO). Post-operative treatment was to be given only in children over 1 year of age at diagnosis who had residual disease or lymph node (LN) involvement. Between 1990 and 1994, 130 consecutive children were registered. In comparison with resectable primaries, these tumours were more commonly abdominal, larger and associated with N-*myc* amplification (NMA). Complete, very good and partial response (CR, VGPR, PR) to CE were, respectively, 1%, 7% and 44%, overall response rate (RR) to two courses of CE and two courses of CAdO was 71%, and the tumour could be removed in all but four of the children. The toxicity was manageable. The 5-year overall survival (OS) and event-free survival (EFS) were, respectively, 88% and 78% with a median follow-up of 38 months. In multivariate analysis, only NMA and LN involvement adversely influenced the outcome, particularly NMA. Children with unresectable NBs and no NMA fared as well as children with resectable ones as OS were, respectively, 95% and 99% and EFS 89% and 91%. Our data show encouraging results in localized but unresectable NBs as 90% of children may be considered as definitely cured, especially those without NMA.

**Keywords:** neuroblastoma; carboplatin; etoposide; N-*myc*

Neuroblastoma (NB) is the most common solid tumour of early childhood (Bernstein et al, 1992). Approximately 50% of patients present with localized tumours (Hartmann et al, 1983; Rosen et al, 1984*a*) and radical surgical excision is generally considered as the main requirement for cure (Evans et al, 1976; Le Tourneau et al, 1985). Primary surgery can be performed in about half of these children and reported survival rates are high (De Bernardi et al, 1995). However, unresectable tumours usually have a poorer outcome, unless secondary radical excision can be performed (Rosen et al, 1984*b*; Haase et al, 1989; Tsuchida et al, 1992). Consequently, the efficacy of primary chemotherapy to allow subsequent resection is of outstanding importance (Garaventa et al, 1993; West et al, 1993). We previously reported the efficacy of the combination of carboplatin and etoposide (CE) in refractory or relapsed NBs (Frappaz et al, 1992) and investigated its relevance as a first line therapy in unresectable NBs.

In 1990, a national prospective study (NBL 90) was initiated, registering all children with localized NBs diagnosed in the institutions of the French Society of Pediatric Oncology (SFOP). The Primary aim was to assess the efficacy and the safety of such chemotherapy as primary treatment in unresectable NBs, including dumbbell tumours. We report herein the results of this treatment strategy.

*Received 20 June 1997*
*Revised 20 November 1997*
*Accepted 27 November 1997*

*Correspondence to:* H Rubie, Unité d' Hémato-Oncologie Pédiatrique, Service de Médecine Infantile B, CHU Purpan, 31059 Toulouse Cédex, France

### PATIENTS AND METHODS

### Patient population

Untreated children aged from 0 to 16 years were eligible. The primary tumour was evaluated using computerized tomography (CT)-scan or magnetic resonance imaging (MRI) as well as metaiodobenzylguanidine (MIBG) scintigraphy. Work-up to eliminate metastatic spread included the skeletal study by MIBG (or a $^{99m}$Tc scan in the absence of MIBG uptake at the primary site), radiograph in infants and extensive bone marrow staging (at least two aspirations and two trephine biopsies). Urinary catecholamines (VMA, HVA and dopamine), serum neuron-specific enolase (NSE), ferritin and lactate dehydrogenase (LDH) levels were measured. The diagnosis of NB was always confirmed by cytological or histological documentation. The primary tumour was staged according to TNM (Beahrs, 1983) and INSS (Brodeur et al, 1993). However, those unresectable NBs did not overlap completely with INSS stage 3 tumours. Indeed, according to our definition of resectability using radiological data, those tumours were not operated first and some of them were INSS stage 2 (i.e. lateral tumours encasing regional organs or vessels and dumbbell tumours). Analysis of the N-*myc* oncogene (Seeger et al, 1985)

This work has been in part reported at the XXIIIth meeting of the SIOP (Rhodes, 1–4 October 1991) and the ASCO meeting (Orlando, 16–18 May 1993).

**Figure 1** Chemotherapy regimen *First period (March 1990–April 1991): Carboplatin (160 mg/m$^2$/d)–Etoposide (100 mg/m$^2$/d) × 5 days. Second period (April 1991–December 1994): Carboplatin (200 mg/m$^2$/d)–Etoposide (150 mg/m$^2$/d) × 3 days. **Each course was to be administered every 21 days or as soon as haematological criteria were met (WBC > $10^9$/l, PMN ≥ 0.5 × $10^9$/l, Platelets > 100 × $10^9$/l). ***Vincristine: maximum dose of 2 mg. N.B. Some newborn received only Vincristine (0.05 mg/kg D1) and Cyclophosphamide (5mg/kg D1–D5)

was recommended for all tumours and amplification (NMA) was defined as the presence of ten copies or more per haploid genome.

### Surgery

Participating institutions were provided with guidelines for surgical procedures. Resectability was defined according to imaging data. Procedures that would have resulted in the removal of major organs were not recommended unless initial chemotherapy had been administered before any attempt of excision. Tumours defined as unresectable were lesions that crossed and infiltrated the mid-line structures, usually encasing large vessels, and tumours that because of size, structure or location were difficult to resect without a high risk of rupture or major surgical complications. All dumbbell tumours were deemed unresectable and were urgently assigned to chemotherapy. Post-operative imaging (CT scan and/or MRI) was required in all patients 1 month after resection. Post-surgical staging was defined on the basis of surgical, pathological reports and post-operative imaging data.

### Chemotherapy

Primary chemotherapy consisted of two courses of CE given as previously described (Frappaz et al, 1992), followed by two courses of vincristine, cyclophosphamide and doxorubicin – CAdO (Figure 1). After April 1991, the dose intensity of CE was decreased because of unacceptable haematological toxicity. Drug doses were always reduced by 30–50% in infants or in children weighing less than 10 kg. After surgery, chemotherapy was indicated in children over 1 year at diagnosis in cases of residual disease and/or lymph node (LN) involvement, or in infants with NMA. Such patients were to receive alternated CE with CAdO for one course each. In patients over 1 year at diagnosis with a persistent macroscopic residue after chemotherapy, a second-look surgical procedure was recommended after post-operative chemotherapy. On the whole, children received a maximum of three courses of each combination.

### Radiotherapy

In case of a persistent macroscopic residue at the end of the treatment, irradiation of the tumour bed was scheduled only in children over 1 year at diagnosis. Doses that ranged from 25 to 35 Gy according to age were delivered in daily fractions of 1.5 to 1.8 Gy each. As of November 1992, locoregional irradiation was recommended for children whose tumour had NMA because of a high

**Table 1** Patient characteristics with localized and unresectable neuroblastoma

| Cases | Unresectable number (%) | Resectable number (%) | *P* |
|---|---|---|---|
| | 130 (41) | 186 (90) | |
| Sex male | 68 (52) | 99 (53) | NS |
| Age (months) | | | |
| Median (range) | 14 (0–192) | | |
| < 12 months | 52 (40) | 100 (54) | 0.01 |
| Site of primary tumour | | | |
| Abdomen, lateral | 55 (42) | 97 (52) | |
| Abdomen, median | 30 (23) | 9 (5) | $10^{-6}$ |
| Mediastinal | 32 (25) | 55 (30) | |
| Pelvic | 10 (8) | 10 (5) | |
| Cervical | 3 (2) | 15 (8) | |
| Dumbbell | 34 (26) | 8 (4) | |
| TNM Stage | | | |
| T1 | 17 (13) | 100 (54) | $10^{-7}$ |
| T2 | 69 (53) | 73 (39) | $10^{-2}$ |
| T3 | 44 (34) | 11 (6) | $10^{-7}$ |
| T5 | 0 | 2 (1) | |
| Initial histology | | | |
| Neuroblastoma | 57/77 (74) | 118/182 (55) | NS |
| MIBG scintigraphy | | | |
| Positive | 118/125 (94) | 127/161 (79) | $2.10^{-4}$ |
| Elevated urinary catecholamine excretion | 111/120 (92) | 94/138 (68) | $10^{-6}$ |
| Abnormal NSE | 95/107 (89) | 85/117 (73) | $2.10^{-3}$ |
| Abnormal ferritin | 15/87 (17) | 11/98 (11) | NS |
| Abnormal LDH | 58/75 (77) | 56/90 (62) | 0.04 |
| N-*myc* analysis | | | |
| ≥ 10 copies | 17/86 (20) | 5/139 (4) | $5.10^{-5}$ |

© Cancer Research Campaign 1998

**Figure 2** Response to chemotherapy induction. CE, carboplatin–etoposide; CR, complete response; VGPR, very good partial response; PR, partial response; NR, no response; PD, progressive disease; NE, not evaluable. *Toxic death (n=1), after cyclophosphamide–vincristine incidence of local relapses, regardless of the age or the quality of surgical excision.

### Evaluation of response to therapy

Response to therapy was assessed according to INRC criteria (Brodeur et al, 1993). The value of tumour response, based on a reduction in volume that was considered more significant than that of urinary catecholamine excretion, was evaluated during induction therapy (every two courses) before and 1 month after surgery, at the end of the treatment, and then at least every 3 months.

### Statistical analysis

To prevent selection bias, all consecutive patients with newly diagnosed localized NB in the participating centres were included in the analysis, whatever the treatment actually administered. The probabilities of survival were calculated from the time of diagnosis to death or last follow-up according to the Kaplan–Meier product-limit method (Fleiss, 1981). In the EFS analysis, disease progression or relapse and death, whatever the reasons, were considered as events. Comparisons between proportions were performed with the $\chi^2$-test corrected for heterogeneity or Fisher's exact test (Peto et al, 1977). Multivariate assessment of EFS times was performed by Cox's proportional hazards model and differences between curves were tested for statistical difference by the log-rank test (Cox, 1977). Potential prognostic factors were included in the multivariate model, provided the number of evaluable patients was sufficient. All tests are two-tailed.

### RESULTS

From March 1990 to December 1994, a total of 337 consecutive children with localized NB were registered in the study. Of those, 21 were excluded because the tumour was a total mature ganglioneuroma. Among the 316 remaining children, 186 underwent primary surgical excision. Therefore, the present analysis concerns 130 patients and reports the outcome of patients as of June, 1997, 30 months after the last patient's inclusion.

### Patient characteristics (Table 1)

The median age was 14 months and 12 children were newborn. Compared with resectable NBs, unresectable tumours were more frequently observed in older children, abdominal site (65%) and

**Table 2** Post-surgical staging according to initial treatment

| TNM post-surgical staging | Initial surgery (n = 186) | Initial chemotherapy (n = 130) | | |
|---|---|---|---|---|
| | | Response (n = 86)* | No response (n = 41)** | Not assessed (n = 3)*** |
| pSI : complete resection, negative nodes (n = 84) | 72 | 11 | 1 | |
| pSII : complete resection, positive nodes (n = 27) | 24 | 3 | 0 | |
| pSIIIa : microscopic residue (n = 124) | 63 | 39 | 20 | 2 |
| pSIIIb : Macroscopic residue ≤ 10% (n = 51) | 15 | 23 | 13 | |
| pSIIIc : macroscopic residue > 10% or biopsy (n = 25) | 12 | 7 | 6 | |

*No surgery, n = 3 (complete remission after chemotherapy n = 1; progressive disease n = 2). **No surgery, n = 1 (progressive disease n = 1). ***Not assessed, n = 3 (no imaging n = 2; toxic death before surgery n = 1).

© Cancer Research Campaign 1998

larger. Large tumours (T3, diameter > 10 cm) were more common in the abdomen (37 out of 85, 43%) than in the chest (6 out of 32, 19%) ($P = 0.02$). Positive MIBG uptake of the primary and elevated biological markers were more frequent in those tumours as well as NMA. Dumbbell tumours were found in 34 children, of whom 27 had a neurological deficit and are described more precisely elsewhere (Plantaz et al, 1996).

### Primary chemotherapy

### *Response (Figure 2)*

Of the 130 children assigned to receive primary chemotherapy, 113 had CE as first courses, according to the protocol. Four of them were not evaluable: two patients had major complications after the first course (intratumoral haemorrhage, $n = 1$ and disseminated candida infection, $n = 1$), and then underwent complete surgical excision; two additional patients had no imaging evaluation. Of the 109 children evaluable after the two courses of CE, 57 (52%) had a response >50% (CR $n = 1$; VGPR $n = 8$; PR $n = 48$), 50 patients did not respond and two progressed while on therapy. Among the 17 children receiving CAdO as first courses, a newborn presenting with a large thoracic and dumbbell tumour died from a massive pulmonary embolism a few days after initiation of chemotherapy, seven had PR and nine failed to respond. After these two first courses, 17 out of 129 children underwent surgical resection, either because of chemotherapy-induced complications ($n = 2$) or because of the physician's decision ($n = 15$). Thus, 112 children received two consecutive courses of chemotherapy according to the protocol; among the 108 evaluable for response after each series of two courses, 21 of the 51 (41%) who did not respond to the first courses responded to the new combination. Consequently, the overall response rate (RR) was 71%, regardless of the type of chemotherapy. Dumbbell tumours responded as well as other primaries (Plantaz et al, 1996). Finally, all but five of the children underwent surgery: one died early, three had progressive disease and died of the disease and one was in CR according to imaging data. As shown in Table 2, of the 125 children evaluable for surgery, 76 (61%) had complete removal of tumour, compared with 159 out of 186 (85%) resectable primaries ($P = 8.10^{-6}$). Among the 49 patients with macroscopic residual disease, 14 underwent a second-look procedure (after two additional courses of chemotherapy in five) leading to a secondary complete resection in eight. Finally, given that the ultimate goal was achievement of CR, primary chemotherapy allowed radical

**Table 3** Toxicity of carboplatin – etoposide.

| | First period* | Second period* | *P* significance |
|---|---|---|---|
| Number of evaluable courses | 111 | 274 | |
| Leucopenia (WBC < $10^9$/l) | | | |
| *n* (%) | 26 (23) | 34 (12) | $7.10^{-3}$ |
| Median duration – days (range) | 4 (1–19) | 5 (1–3) | NS |
| Neutropenia (PMN < $0.510^9$/l) | | | |
| *n* (%) | 76 (67) | 123 (45) | $10^{-4}$ |
| Median duration – days (range) | 7 (1–15) | 7 (1–17) | NS |
| Thrombocytopenia (Plts < $5010^9$/l) | | | |
| *n* (%) | 57 (51) | 84 (31) | $10^{-4}$ |
| Median duration – days (range) | 2 (1–14) | 3 (1–13) | NS |
| Transfusions | | | |
| RBC | | | |
| *n* (%) | 52 (47) | 75 (27) | $2.10^{-4}$ |
| Median number (range) | 1 (1–3) | 1 (1–3) | NS |
| Platelets | | | |
| *n* (%) | 42 (38) | 66 (24) | $2.10^{-3}$ |
| Median number (range) | 1 (1–6) | 1 (1–5) | NS |
| Fever > 38°C | | | |
| *n* (%) | 38 (34) | 63 (23) | $2.10^{-2}$ |
| Median duration – days (range) | 4 (1–30) | 2 (1–10) | $4.10^{-3}$ |
| Documented infection | | | |
| *n* (%) | 12 (11) | 32 (12) | NS |
| Intravenous antibiotics | | | |
| *n* (%) | 17 (15) | 36 (13) | NS |
| Median duration – days (range) | 12 (2–40) | 8 (1–28) | NS |
| Alteration of creatinine clairance (drop ≥ 30% of initial value) | | | |
| *n* (%) | 4/33 (12) | 3/48 (6) | NS |
| Alteration of audiometry | | | |
| *n* (%) | 0/28 | 0/23 | |
| Miscellaneous | 0 | 2 (1)** | |

*First period from March 1990 to April 1991. Second period from April 1991 to December 1994 (schedule and doses are detailed Figure 1).
**Anaphylaxis after etoposide.

© Cancer Research Campaign 1998

**Figure 3**: Event-free survival (EFS) of children with unresectable and resectable neuroblastoma (NB). — EFS children with resectable NB ($n$=186) = 88 ± 3%.- - - EFS children with unresectable NB ($n$=130) = 78 ± 6%

excision to be performed in 67% of patients with unresectable tumour at diagnosis.

### *Toxicity*

The main data are listed in Table 3, including primary and/or post-operative chemotherapy. The toxicity of the CE regimen was mainly haematological and manageable. During the first period of the study, more than half of the patients experienced WHO grade 4 cytopenia. Transfusions were necessary in nearly half of the patients. Four major chemotherapy-related complications were observed: one child had a life-threatening infection after the first course, then underwent surgery and received four subsequent courses without any complication; another one had an intracerebral haemorrhage associated with CE-related thrombocytopenia and is alive without sequelae; the last two patients developed virus C hepatitis after transfusion. Therefore, the total doses of this combination were subsequently reduced in April 1991. Among the 274 courses given thereafter, the incidence of neutropenia, fever, thrombocytopenia and transfusions was significantly lowered and no major complication was observed. In addition, the RR was not compromised (respectively 58% and 50% for the first and the second period). Regarding renal toxicity, the reduction in creatinine clearance was transient. The toxicity of CAdO was haematological. Briefly, among the 215 evaluable courses, red blood cell and platelet transfusions were given in respectively 49% and 11% of patients, WHO grade 4 neutropenia was observed in 70% of patients and documented infection in 27%, and intravenous antibiotics were administered in 45% of children. All the complications were easily manageable. However, granulocyte colony-stimulating factor has been administered after 30% of the courses, and that may have lowered neutropenia incidence and its duration.

### Post-operative treatment

### *Chemotherapy*

Among the 113 children with either a complete resection and positive LN or residual disease after surgery (Table 2), 65 were over 1 year at diagnosis. Fifty-seven received two post-operative courses

**Table 4** Prognostic factors

| | Event/patient | Univariate analysis | |
|---|---|---|---|
| | No (EFS) | *P*-value (log rank) | Hazard ratio |
| Sex | | | |
| Male | 15/68 (77) | | |
| Female | 13/62 (78) | NS | |
| Age | | | |
| 0–12 months | 5/52 (90) | | |
| ≥ 12 months | 23/78 (69) | $7.10^{-3}$ | 2.75 (1.1–3.8) |
| Site of primary tumour | | | |
| Abdominal | 24/85 (72) | | |
| Non abdominal | 4/45 (91) | $2.10^{-2}$ | 3.6 (1.2–10.4) |
| Abdominal median | 9/30 (68) | | |
| Right | 10/26 (49) | | |
| Left | 5/29 (81) | $2.10^{-2}$ | |
| Size of primary tumour | | | |
| T1 | 2/17 (88) | | |
| T2 | 6/69 (91) | | |
| T3 | 20/44 (54) | $10^{-5}$ | 5.6 (2.5–12.7) |
| Histological lymph node invasion | | | |
| No | 5/72 (92) | | |
| Yes | 17/47 (62) | $3.10^{-5}$ | 6.17 (2.3–16.6) |
| MIBG uptake | | | |
| Positive | 23/118 (80) | | |
| Negative | 5/7 (29) | $7.10^{-4}$ | 4.65 (1.7–12.2) |
| Initial cytology or histology | | | |
| Neuroblastoma | 14/57 (75) | | |
| Ganglioneuroblastoma | 3/20 (82) | NS | |
| Urinary catecholamines | | | |
| VMA/HVA ≥ 1 | 6/53 (91) | | |
| VMA/HVA < 1 | 22/74 (68) | $5.10^{-3}$ | 2.68 (2.1–3.4) |
| Dopamine | | | |
| <2000 | 9/64 (86) | | |
| 2000–3000 | 3/11 (73) | NS | |
| >3000 | 15/45 (64) | $10^{-2}$ | 2.15 (1.1–6.6) |
| NSE | | | |
| Normal | 0/12 (100) | | |
| Abnormal > 2 N | 20/75 (74) | NS | |
| Ferritin | | | |
| Normal | 15/73 (80) | | |
| Abnormal > 2 N | 4/11 (70) | NS | |
| LDH | | | |
| Normal | 1/17 (94) | | |
| Abnormal > 2 N | 7/15 (53) | $10^{-2}$ | 8 (1.6–39.6) |
| N-*myc* amplification | | | |
| < 10 copies | 7/69 (89) | | |
| ≥ 10 copies | 10/17 (41) | $7.10^{-7}$ | 7.09 (2.8–18.1) |
| Response to initial chemotherapy | | | |
| CR/VGPR/PR | 21/83 (75) | | |
| NR/PD | 6/45 (84) | NS | |
| Results of surgery | | | |
| CR | 12/76 (85) | | |
| VGPR + PR | 12/49 (75) | NS | |
| Final result of therapy | | | |
| CR | 12/93 (87) | | |
| No CR | 13/36 (65) | $10^{-4}$ | 3.8 (1.8–8) |
| Protocol compliance | | | |
| Good | 24/106 (78) | | |
| Violation | 4/21 (80) | NS | |

| | Multivariate analysis* | |
|---|---|---|
| | *P*-value | Hazard ratio |
| N-*myc* amplification | $2.10^{-4}$ | 7.8 (2.6–23.2) |
| Histological lymph node involvement | $6.10^{-3}$ | 6.1 (1.7–22.3) |

*Analysis performed in patients with age, size and site of the primary, MIBG uptake, histological lymph node involvement and N-*myc* amplification all evaluable ($n$ = 75).

 © Cancer Research Campaign 1998

of chemotherapy according to the protocol, and in eight cases no further therapy was given (poor general condition, *n* = 2 or protocol violations, *n* = 6).

### *Radiotherapy*

After the amendment, radiotherapy was given in four children in CR (two infants), because NMA was documented. Among the 36 children with a persistent macroscopic residue at the end of treatment, 15 were under 1 year of age and irradiation was omitted in all but two (protocol violations). Conversely, of 21 older patients, eight were not irradiated according to the physician's judgement. Irradiation was delivered in 13, with final tumour doses ranging from 20 to 45 Gy (mean tumour dose 32.32 ± 6.28 Gy) and fields including vertebrae 1–11 (mean 4,6). No immediate toxicity was reported. Owing to the protocol, radiation therapy was finally avoided in 26 out of 49 patients (53%) who had a post-operative macroscopic residue.

### Outcome

Among the 130 children, the disease status at the end of treatment was CR in 93 (72%), VGPR in 27, PR in one, NR in one and PD in seven. With the present follow-up (FU), an event occurred in 28 (21%) children. Two patients died of treatment-related toxicity, either after chemotherapy (*n* = 1) or after subsequent surgery (*n* = 1) and one of an unexplained cause (18 months after diagnosis). Seven patients developed PD, of whom one is alive and disease-free after salvage therapy including high-dose chemotherapy (HDC) and bone marrow transplantation (BMT). Eighteen children experienced a relapse that was either local (*n* = 13), metastatic (*n* = 3) or combined (*n* = 2), at a median time of 7 months after diagnosis (range 2–27 months).

As of June 1997, 113 out of 130 children are alive, with a median FU of 38 months (range 30–84 months). Eight patients developed severe sequelae (neurological and/or orthopaedic), all arising in dumbbell tumours exhibiting symptoms (Plantaz et al, 1996). One child developed major acute renal failure after secondary surgery, leading to kidney transplantation, and is still alive in remission. One child presented with Ewing's sarcoma 3 years after initial diagnosis and is still in remission 30 months later.

**Figure 4** Event-free survival (EFS) of children with no N-*myc* amplification according to primary resectability. — Resectable NBs (*n*=134). EFS = 91 ± 4%. - - - Unresectable NBs (*n*=69). EFS=89 ± 8%. Log rank = 0.6

### Survival and prognostic factors

As shown in Figure 3, the projected overall survival (OS) and event-free survival (EFS) rates at 5 years were respectively 88% ± 6% and 78% ± 6% with a median follow-up of 38 months (range 30–84 months). Table 4 shows the univariate and multivariate analysis of all the usual prognostic factors. Despite infants' treatment being reduced, their outcome was better than older children. In the univariate analysis, a large primary influenced adversively the outcome, as well as abdominal site, histologically proven LN involvement, low VMA/HVA ratio, elevated LDH and NMA. When the established prognostic factors were combined with NMA in the Cox regression model, NMA was the most powerful indicator of poor outcome. EFS was far better in the 69 children with less than ten copies compared with the 17 with NMA (89% vs 41%) (log rank = $7.10^{-7}$). Subsequent relapse was observed in 10 out of 17 children with NMA, either local (*n* = 5), metastatic (*n* = 3) or combined (*n* = 2), from 3 to 17 months after diagnosis. All of them ultimately died of disease despite salvage therapy including HDC followed by BMT (*n* = 4). As shown in Figure 4, children with unresectable NB and no NMA fared as well as children with resectable tumour, as OS were respectively 95% ± 5% and 99% ± 5%, and EFS were 89% ± 8% and 91% ± 4% (log rank = 0.7). The only other significant prognostic factor was histological LN invasion. However, in children without NMA, the outcome of those with positive (*n* = 25) and negative LN (*n* = 42) was comparable (EFS respectively 82% ± 16% and 95% ± 7%, *P* = 0.12).

### DISCUSSION

Unresectable NB usually carries a poor prognosis as achieving EFS greater than 50% has proven difficult, despite introducing chemotherapy in the early 1980s (Evans et al, 1984; Rosen et al, 1984*b*). Encouraging results have recently been reported using more intensive regimens with improved EFS to 60–70% (Nitschke et al, 1991; Garaventa et al, 1993; West et al, 1993; Castel et al, 1995). However, such efficacy should be balanced with the risk of treatment-related sequelae as most of these regimens included high cumulative doses of alkylating agents, doxorubicin and *cis* platinum and sometimes radiotherapy (Castleberry et al, 1991). This led us to design a prospective, multicentric study using modern tools for assessment and evaluating new and presumably less toxic treatment schedules in localized but unresectable NBs.

Biased selection was avoided by registering all consecutive cases of localized NBs. Although our definition of unresectable tumours relied on imaging and not operative findings, patient characteristics were similar to those of other published series (Garaventa et al, 1993; West et al, 1993; Castel et al, 1995).

The therapeutic strategy chosen provided good results as nearly 90% of patients are long-term survivors and probably cured. This result should be emphasized as it differs from previous reports. Two reasons may account for our encouraging results. First, localized tumours were rigourously selected according to INSS recommendations throughout work-up that included initial MIBG scintigraphy and extensive bone marrow staging to eliminate metastatic dissemination. Such extensive staging has not been performed in all children in the most recent studies (Garaventa et al, 1993; West et al, 1993; Castel et al, 1995), which may have included some cases with undetected metastatic disease. As a matter of fact, many of the patients described in these studies experienced disseminated recurrences, as opposed to 4% of metastatic relapses (28% of the relapses) in our series. Second, most authors agree that the degree of surgical excision in localized NB will influence outcome (Evans et al, 1976; Le Tourneau et al, 1985; Haase et al, 1989; Garaventa et al, 1993). Consequently, primary chemotherapy permitting radical surgery, and perhaps also post-operative treatment, may exert a major impact on outcome. Similar findings from other groups support the idea that an increase in treatment intensity improves EFS in children with extensive local and regional NB (Castleberry et al, 1991; Garaventa et al, 1993; West et al, 1993; Castel et al, 1995).

The efficacy of CE combination has been previously reported (Frappaz et al, 1992). The present study confirms its efficacy, when combined with CAdO as the overall tumour response rate was over 70%, and 97% of the patients could undergo surgical resection. Although the incidence of persistent macroscopic residual disease was still higher than in resectable NBs, the EFS rate of those children was still good (75% vs 85%). Moreover, this chemotherapy combination appears appropriate in dumbbell tumours as neurological recovery was observed in 90% of cases without laminectomy (Plantaz et al, 1996). Amazingly, response to chemotherapy was not predictive of subsequent outcome. One may suggest that NBs considered as NRs included tumours with 10–49% shrinkage, which could be sufficient for radical excision; the similar incidence of macroscopic residue in responding (30 out of 83 = 36%) and non-responding patients (19 out of 40 = 47%) might support this hypothesis. Furthermore, NRs have probably occurred in more mature tumours, which might have a lower risk of subsequent relapse. The toxicity of the chemotherapy regimen used was acceptable. The only toxicity-related death occurred after small doses of cyclophosphamide–vincristine, and the fatal pulmonary embolism could have been caused by the large thoracic tumour as well. Although the chemotherapy regimen was short and total cumulative doses of drugs were low (maximum doses of carboplatin = 1.8–2.4 g $m^{-2}$ according to the period of study, cyclophosphamide = 4.5 g $m^{-2}$, doxorubicin = 180 mg $m^{-2}$), there is still concern about the possible late side-effects and the long-term evaluation of audiometry and renal function is needed to confirm the optimal cost–benefit ratio.

The significance of prognostic factors in such a population is still controversial. Age is one of the most powerful (Evans et al, 1976; Hartmann et al, 1983; Garaventa et al, 1993). In our study, despite the de-escalation of post-operative treatment in infants, EFS was better than in older children. Given these results, less intensive chemotherapy will be prospectively evaluated in infants in the next trial. We confirm that abdominal primaries had a significantly worse outcome than non-abdominal sites. The adverse outcome of patients with a right-sided abdominal tumour may be due to the more difficult surgical resection in that site. Children with large tumours (T3) fared significantly worse than those with small ones and it should be pointed out that 84% of those were abdominal. Actually, the most relevant finding in this study was the extremely powerful significance of NMA in this population. Correlations between NMA and the usual adverse prognostic factors have been firmly established (Seeger et al, 1985; Brodeur et al, 1992), and we have recently confirmed its negative influence on outcome in localized NBs (Rubie et al, 1997). Among the 86 tumours analysed before any adjuvant treatment, NMA was found in 20% of the tumours and correlated with well-known adverse prognostic factors such as age, site and the size of the primary and elevated biological markers. Furthermore, once they relapse, these children cannot be salvaged by any kind of second-line treatment as all died a few months later. Our finding differs from the POG's data (Cohn et al, 1995), but their study did not include stage C tumours. Indeed, this selection may have reduced the incidence of amplified tumours as in our series of localized NBs, 17 out of 22 children with NMA had an unresectable primary. In the multivariate analysis, LN invasion ranked second. The poor prognostic value of LN involvement has been pointed out by some authors (Ninane et al, 1982; Castleberry et al, 1991; De Bernardi et al, 1995) but not necessarily confirmed by others (Rosen et al, 1984*c*; Matthay et al, 1989). In the present series, LN invasion had an adverse effect on outcome, but this predictive value was no more significant in patients without NMA, underlining the significance of such genetic alteration in patients with regional disease.

In conclusion, our data show excellent results in localized but unresectable NBs. The combination of CE followed by CAdO appears to be the appropriate primary treatment in children with such tumours, including dumbbell lesions. N-*myc* amplification, arising in 20% of these tumours, is the most powerful prognostic factor, and an innovative approach is warranted in this subset of patients. With this treatment strategy, patients with unresectable NB and without NMA fared as well as those with resectable primary and do not need stage 4 strategies.

### ACKNOWLEDGEMENT

This work was supported by the Association pour la Recherche contre le Cancer.

### REFERENCES

Beahrs OH (1983) American Joint Committee. Neuroblastoma. In *Manual of Staging of Cancer*, Myers MH (eds), pp. 237–239. Lippincott: Philadelphia

Bernstein ML, Leclerc JM, Bunin G, Brisson L, Robison L, Shuster J, Byrne T, Gregory D, Hill G, Dougherty G, Scriver C, Lemieux B, Tuchman M and Woods WG (1992) A population-based study of neuroblastoma incidence, survival, and mortality in North America. *J Clin Oncol* **10**: 323–329

Brodeur GM, Azar C, Brother M, Hiemstra J, Kaufman B, Marshall H, Moley J, Scavarda N, Schneider S, Wasson J, White P, Seeger RC, Look T and Castleberry RP (1992) Neuroblastoma: effect of genetic factors on prognosis and treatment. *Cancer* **70**: 1685–1694

Brodeur GM, Pritchard J, Berthold F, Carlsen NLT, Castel V, Castleberry RP, De Bernardi B, Evans AE, Favrot M, Hedborg F, Kaneko M, Kemshead J, Lampert F, Lee Rej, Look T, Pearson ADJ, Philip T, Roald B, Sawada T, Seeger RC, Tschuda Y and Voute PA (1993) Revisions of the international criteria for neuroblastoma diagnosis, staging and response to treatment. *J Clin Oncol* **11**: 1466–1477

Castel V, Badal MD, Bezanilla JL, Llombart A, Ruiz-Jimenez JI, Sanchez de Toledo J, Melero C and Mulet J (1995) Treatment of stage III neuroblastoma with emphasis on intensive induction chemotherapy: a report from the neuroblastoma group of the Spanish Society of Pediatric Oncology. *Med Pediatr Oncol* **24**: 29–35

Castleberry RP, Kun LE, Shuster JJ, Altshuler G, Smith IE, Nitschke R, Wharam M, McWilliams N, Joshi V and Hayes FA (1991) Radiotherapy improves the outlook for patients older than 1 year with pediatric oncology group stage C neuroblastoma. *J Clin Oncol* **9**: 789–795.

Cohn SL, Look AT, Joshi VV, Holbrook T, Salwen H, Chagnovich D, Chesler L, Rowe ST, Valentine MB, Komuro H, Castleberry RP, Bowman LC, Rao PV, Seeger RC and Brodeur GM (1995) Lack of correlation of N-*Myc* gene amplification with prognosis in localized neuroblastoma: a pediatric oncology group study. *Cancer Res* **55**: 721–726.

Cox DR (1977) *The Analysis of Binary Data*. Chapman & Hall: London

 © Cancer Research Campaign 1998

De Bernardi B, Conte M, Mancini A, Donfrancesco A, Alvisi P, Toma P, Casale F, Montezemolo LC, Cornelli PE, Carli M, Tonini GP, Pession A, Giaretti W, Garaventa A, Marchese N, Magillo P, Nigro M, Kotitsa Z, Tamaro P, Tamburini A, Rogers D and Bruzzi P (1995) Localized resectable neuroblastoma: results of the second study of the Italian cooperative group for neuroblastoma. *J Clin Oncol* **13**: 884–893

Evans AE, Albo V, D'Angio GJ, Finklesteinjz, Leiken S, Santulli T, Weiner J and Hammond GD (1976) Factors influencing survival of children with non metastatic neuroblastoma. *Cancer* **38**: 661–666

Evans AE, D'Angio GH and Koop CE (1984) The role of multimodal therapy in patients with local and regional neuroblastoma. *J Pediatr Surg* **19**: 77–80

Evans AE, D'Angio GH, Sather HN, De Lorimer AA, Dalton A, Ungerleider RS, Finklestein JZ and Hammond GD (1990) A comparison of four staging systems for localized and regional neuroblastoma: a report from the Childrens Cancer Study Group. *J Clin Oncol* **8**: 678–688

Fleiss JI (1981) *Statistical Methods for Rates and Proportions*, 2nd edn. Wiley: New York

Frappaz D, Michon J, Hartmann O, Bouffet E, Lejars O, Rubie H, Gentet JC, Chastagner P, Sariban E, Brugieres L, Zucker JM, Lemerle J and Philip T (1992) Etoposide and carboplatin in neuroblastoma: a French Society of Pediatric Oncology Phase II study. *J Clin Oncol* **10**: 1592–1601

Garaventa A, De Bernardi B, Pianca C, Donfrancesco A, Montezemolo LC, Di Tullio MT, Bagnulo S, Mancini A, Carli M, Pession A, Arrighini A, Di Cataldo A, Tamaro P, Iasonni V, Tacone A, Rogers D and Boni L (1993) Localized but unresectable neuroblastoma: treatment and outcome of 145 cases. *J Clin Oncol* **11**: 1770–1779

Haase GM, Wong KY, De Lorimier AA, Sather HN and Hammond GD (1989) Improvement in survival after excision of primary tumour in stage III neuroblastoma. *J Pediatr Surg* **24**: 194–200

Hartmann O, Scopinaro M, Tournade MF, Sarrazin D and Lemerle J (1983) Neuroblastomes traités à l'Institut Gustave Roussy de 1975 à 1979. Cent soixante treize cas. *Arch Fr Pédiatr* **40**: 15–21

Le Tourneau JN, Bernard JL, Hendren WH and Carcassonne M (1985): Evaluation of the role of surgery in 130 patients with neuroblastoma. *J Pediatr Surg* **20**: 244–249

McGuire WA, Simmons D, Grosfeld JL and Baehner RL (1985) Stage II neuroblastoma. Does adjuvant irradiation contribute to cure? *Med Pediatr Oncol* **13**: 117–121

Matthay KK, Sather HN, Seeger RC, Haase GM and Hammond GD (1989) Excellent outcome of stage II neuroblastoma is independent of residual disease and radiation therapy. *J Clin Oncol* **7**: 236–244

Ninane J, Pritchard J, Jones PHM, Mannjr and Malpas JS (1982) Stage II neuroblastoma. Adverse prognostic significance of lymph node involvement. *Arch Dis Child* **57**: 438–442

Nitschke R, Smith EI, Altshuler G, Altmiller D, Shuster J, Green A, Castleberry RP, Hayes FA, Golembe B and Ducos R (1991) Post operative treatment of non metastatic visible residual neuroblastoma: a Pediatric Oncology Group Study. *J Clin Oncol* **9**: 1181–1188

Peto R, McPherson K and Peto J (1977) Design and analysis of clinical trials requiring prolonged observation of each patient. *Br J Cancer* **35**: 1–39

Plantaz D, Rubie H, Michon J, Mechinaud F, Coze C, Chastagner P, Frappaz D, Gigaud M, Passagia JM and Hartmann O (1996) The treatment of neuroblastoma with intraspinal extension with chemotherapy followed by surgical removal of residual disease. A prospective study of 42 cases. Results of the NBL 90 study of the French Society of Pediatric Oncology. *Cancer* **78**: 311–319

Rosen EM, Cassady JR, Frantz CN, Kretschmar C, Levey R and Sallan SE (1984*a*) Neuroblastoma: The Joint Center for Radiation Therapy/Dana Farber Cancer Institute/Children's Hospital experience. *J Clin Oncol* **2**: 719–732

Rosen EM, Cassady JR, Frantz CN, Kretschmar C, Levey R and Sallan SE (1984*b*) Improved survival in neuroblastoma using multimodality therapy. *Radiother Oncol* **2**: 189–200

Rosen EM, Cassady JR, Kretschmar C, Frantz CN, Levey R and Sallan SE (1984*c*) Influence of loco-regional lymph node metastases on prognosis in neuroblastoma. *Med Pediatr Oncol* **12**: 260–263

Rubie H, Hartmann O, Michon J, Frappaz D, Coze C, Chastagner P, Baranzelli MC, Plantaz D, Avet Loiseau H, Benard J, Delattre O, Favrot M, Peyroulet MC, Thyss A, Perel Y, Bergeron C, Courbon-Collet B, Vannier JP, Lemerle J and Sommelet D (1997) Localized neuroblastoma: N-*Myc* gene amplification is a major prognostic factor. Results of the French NBL 90 study. *J Clin Oncol* **15**: 1171–1182

Seeger RC, Brodeur GM, Sather HN, Dalton A, Siegel SE, Wong KY and Hammond GD (1985) Association of multiple copies of N-*Myc* oncogene with rapid progression of neuroblastomas. *N Engl J Med* **318**: 111–116

Tsuchida Y, Yokoyama J, Kaneko M, Uchino JI, Iwafuchi M, Makino SI, Matsuyama S, Takayashi H, Okabe I, Hashizume K, Hayashi A, Nakada K, Yokoyama S, Nishihira H, Sasaki S, Sawada T, Nagahara N and Okada A (1992) Therapeutic significance of surgery in advanced neuroblastoma: A report from the Study Group of Japan. *J Pediatr Surg* **27**: 616–622

West DC, Shamberger RC, Macklis RM, Kozakewich HPW, Wayne AS, Kreissman SG, Korf BR, Lavally B and Grier HE (1993) Stage III neuroblastoma over 1 year at diagnosis: improved survival with intensive multimodality therapy including multiple alkylating agents. *J Clin Oncol* **11**: 84–90

© Cancer Research Campaign 1998


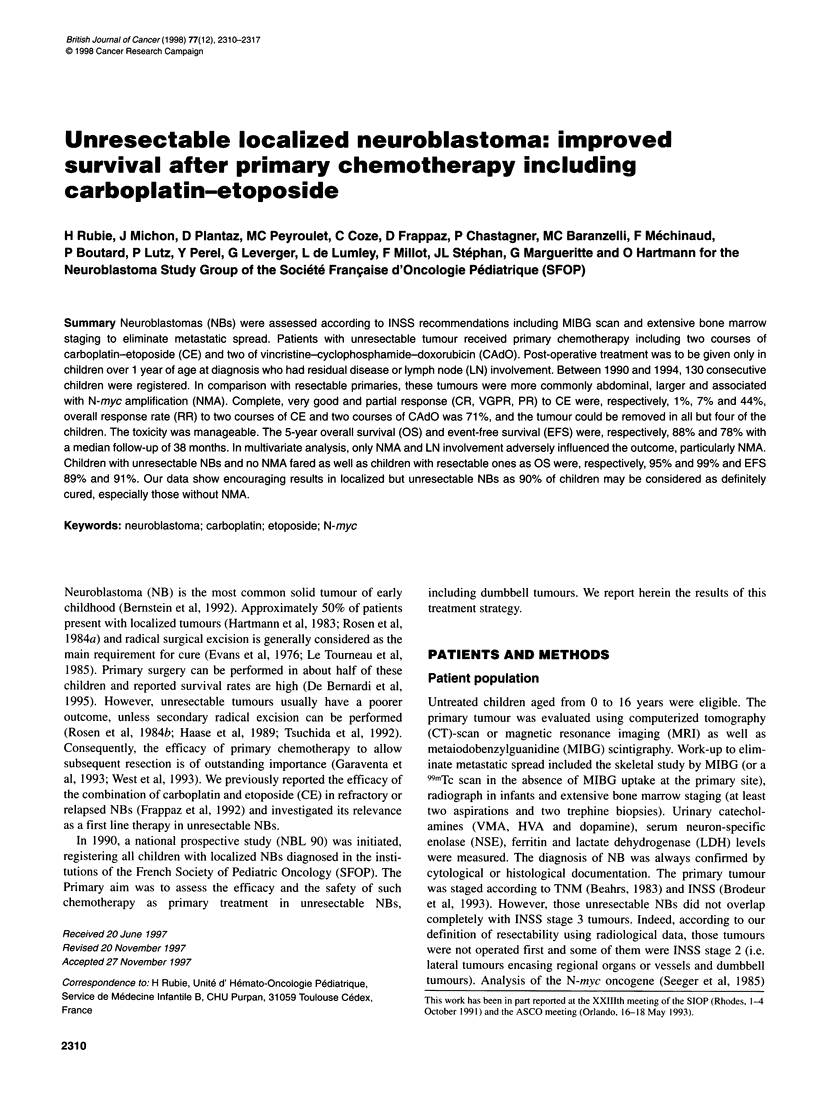

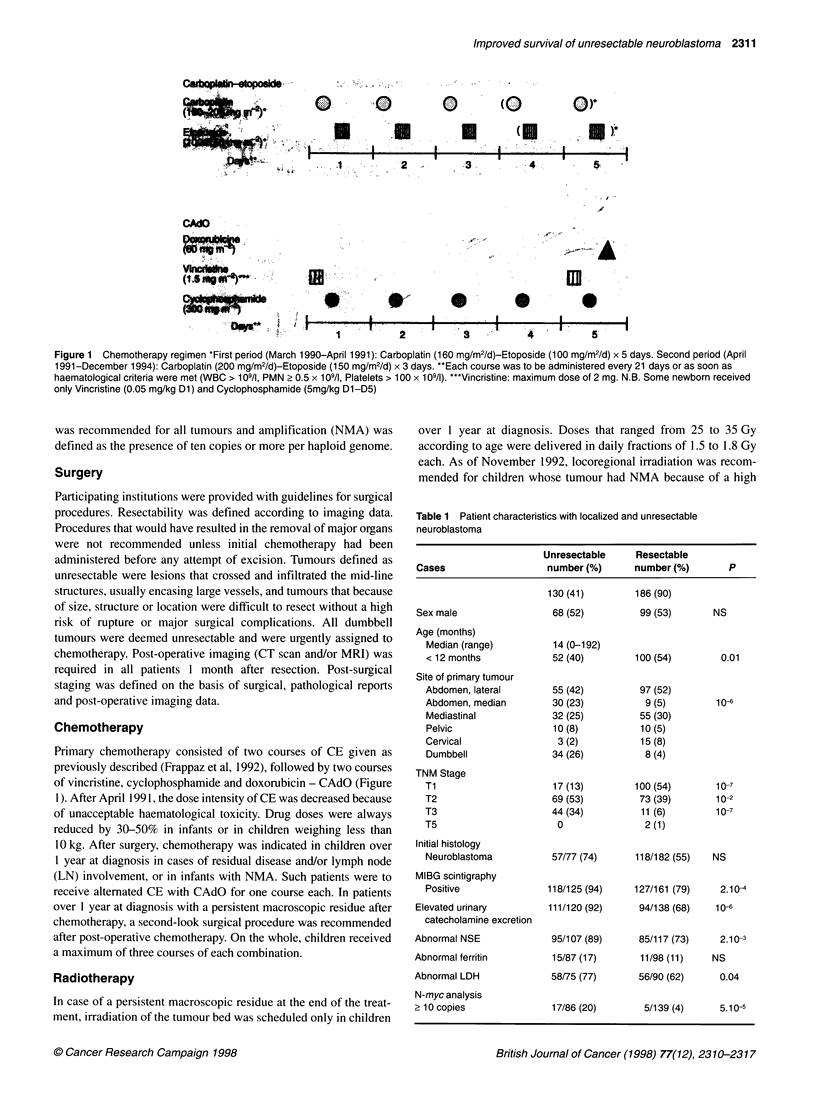

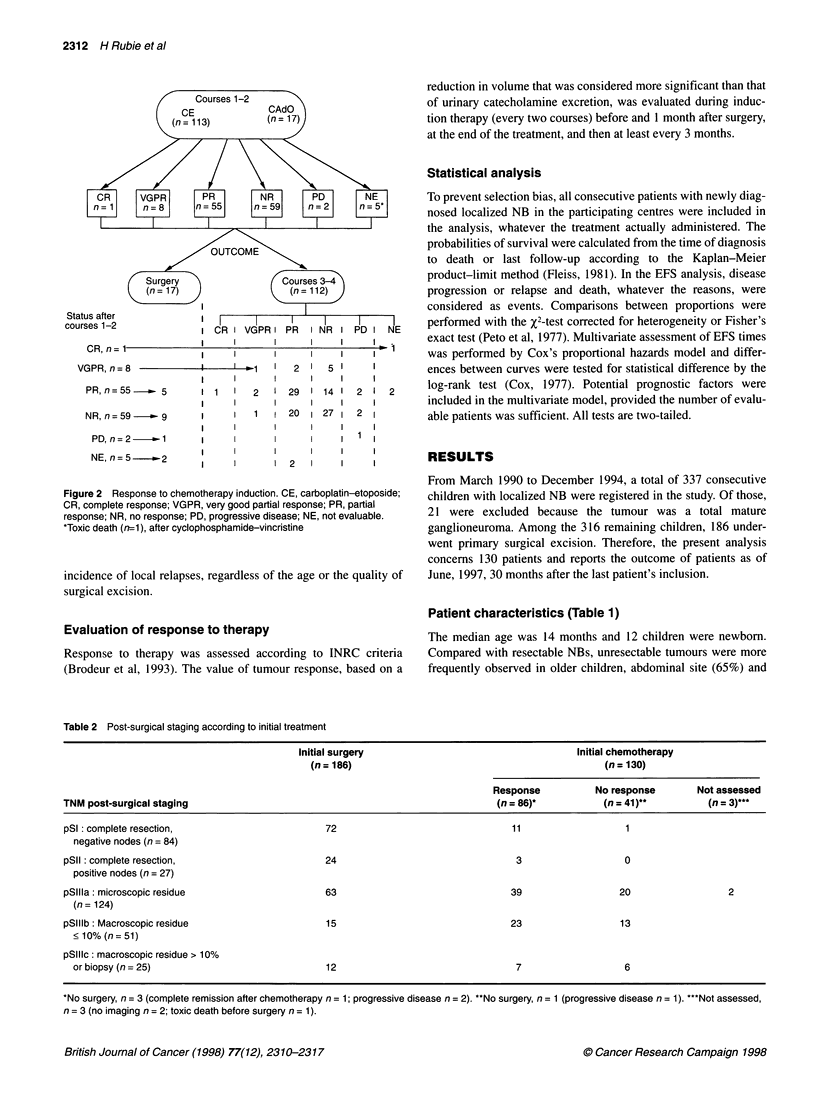

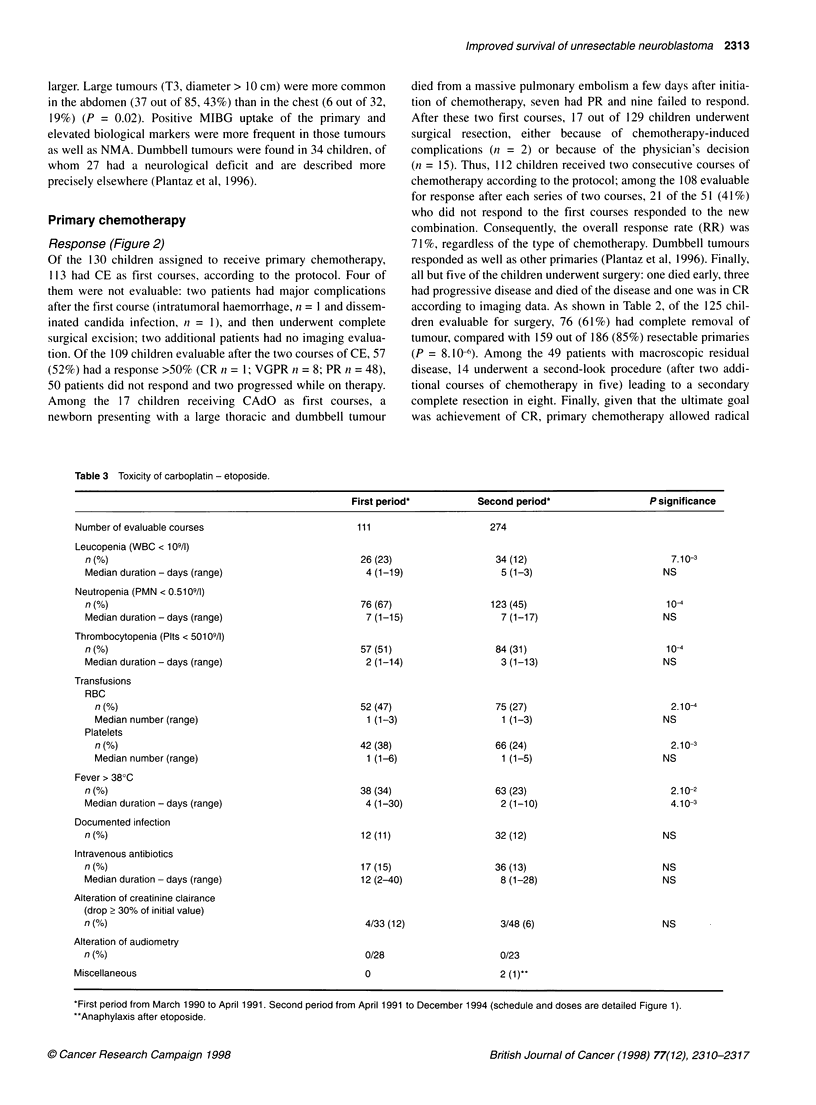

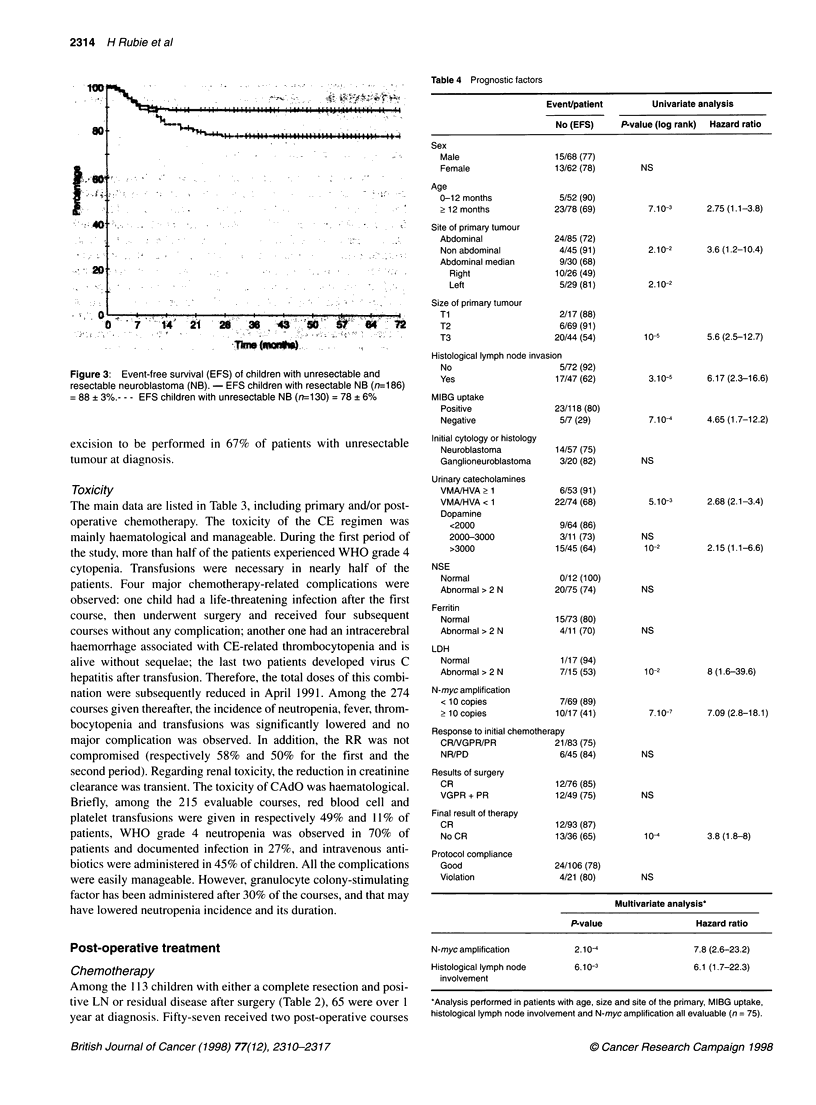

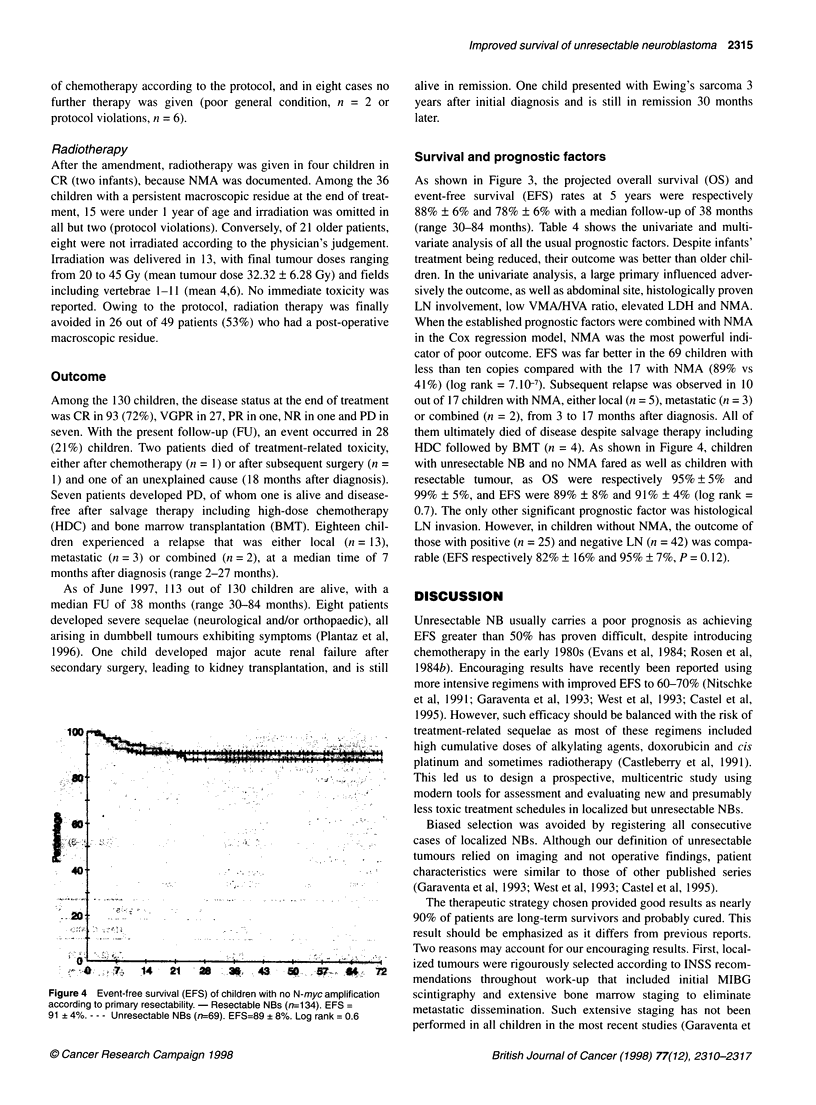

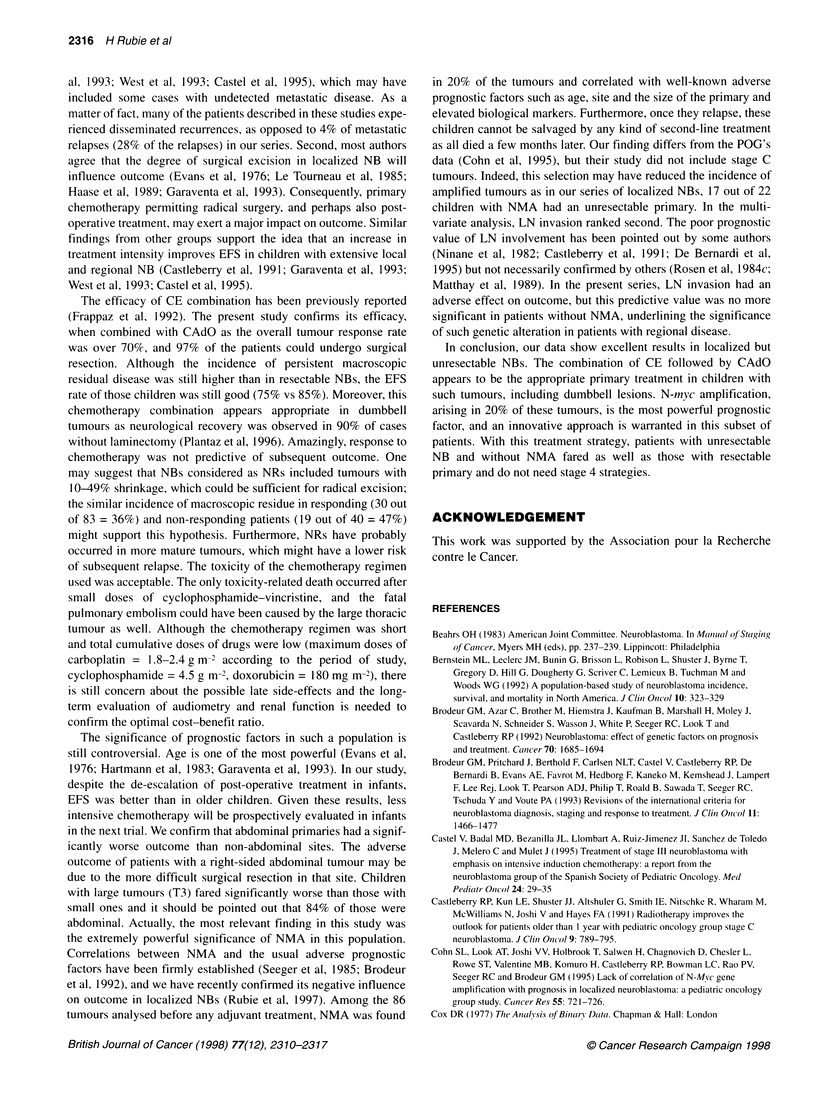

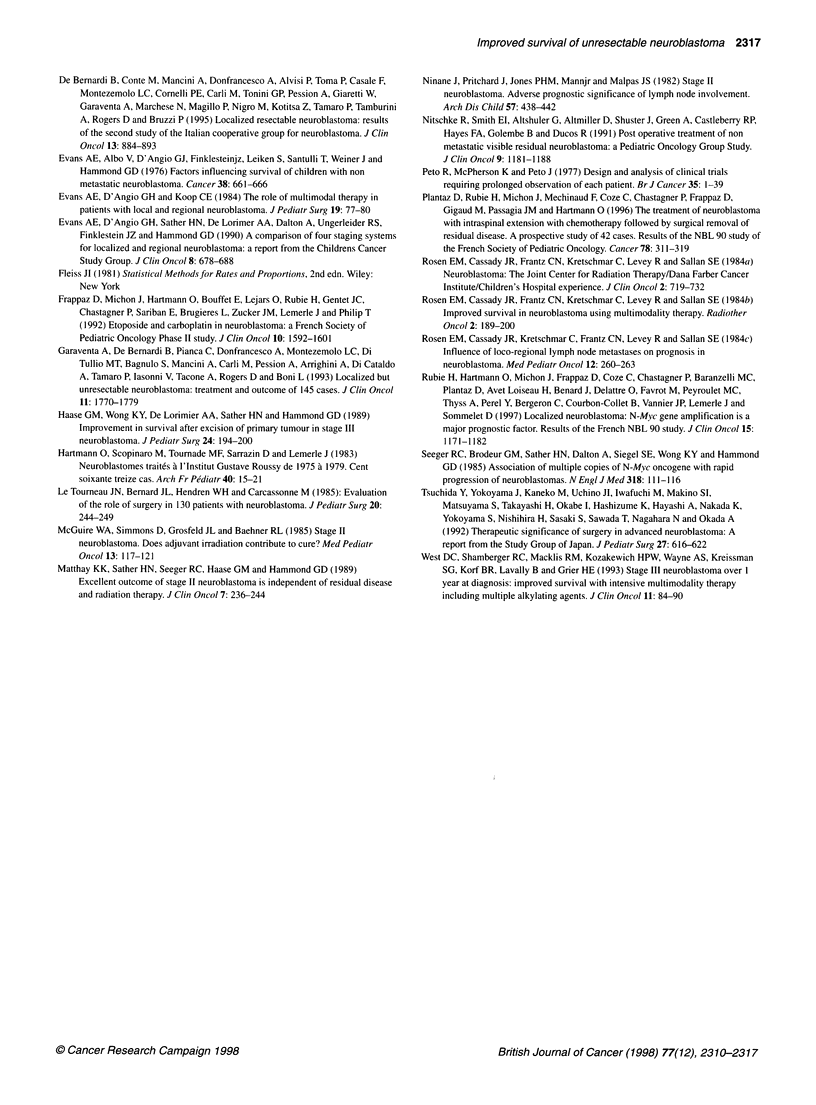


## References

[OCR_00985] Brodeur G. M., Azar C., Brother M., Hiemstra J., Kaufman B., Marshall H., Moley J., Nakagawara A., Saylors R., Scavarda N. (1992). Neuroblastoma. Effect of genetic factors on prognosis and treatment.. Cancer.

[OCR_01002] Castel V., Badal M. D., Bezanilla J. L., Llombart A., Ruiz-Jiménez J. I., Sánchez de Toledo J., Melero C., Mulet J. (1995). Treatment of stage III neuroblastoma with emphasis on intensive induction chemotherapy: a report from the Neuroblastoma Group of the Spanish Society of Pediatric Oncology.. Med Pediatr Oncol.

[OCR_01011] Castleberry R. P., Kun L. E., Shuster J. J., Altshuler G., Smith I. E., Nitschke R., Wharam M., McWilliams N., Joshi V., Hayes F. A. (1991). Radiotherapy improves the outlook for patients older than 1 year with Pediatric Oncology Group stage C neuroblastoma.. J Clin Oncol.

[OCR_01014] Cohn S. L., Look A. T., Joshi V. V., Holbrook T., Salwen H., Chagnovich D., Chesler L., Rowe S. T., Valentine M. B., Komuro H. (1995). Lack of correlation of N-myc gene amplification with prognosis in localized neuroblastoma: a Pediatric Oncology Group study.. Cancer Res.

[OCR_01028] De Bernardi B., Conte M., Mancini A., Donfrancesco A., Alvisi P., Tomà P., Casale F., Cordero di Montezemolo L., Cornelli P. E., Carli M. (1995). Localized resectable neuroblastoma: results of the second study of the Italian Cooperative Group for Neuroblastoma.. J Clin Oncol.

[OCR_01039] Evans A. E., Albo V., D'Angio G. J., Finklestein J. Z., Leiken S., Santulli T., Weiner J., Hammond G. D. (1976). Factors influencing survival of children with nonmetastatic neuroblastoma.. Cancer.

[OCR_01042] Evans A. E., D'Angio G. J., Koop C. E. (1984). The role of multimodal therapy in patients with local and regional neuroblastoma.. J Pediatr Surg.

[OCR_01046] Evans A. E., D'Angio G. J., Sather H. N., de Lorimier A. A., Dalton A., Ungerleider R. S., Finklestein J. Z., Hammond G. D. (1990). A comparison of four staging systems for localized and regional neuroblastoma: a report from the Childrens Cancer Study Group.. J Clin Oncol.

[OCR_01056] Frappaz D., Michon J., Hartmann O., Bouffet E., Lejars O., Rubie H., Gentet J. C., Chastagner P., Sariban E., Brugiere L. (1992). Etoposide and carboplatin in neuroblastoma: a French Society of Pediatric Oncology phase II study.. J Clin Oncol.

[OCR_01062] Garaventa A., De Bernardi B., Pianca C., Donfrancesco A., Cordero di Montezemolo L., Di Tullio M. T., Bagnulo S., Mancini A., Carli M., Pession A. (1993). Localized but unresectable neuroblastoma: treatment and outcome of 145 cases. Italian Cooperative Group for Neuroblastoma.. J Clin Oncol.

[OCR_01070] Haase G. M., Wong K. Y., deLorimier A. A., Sather H. N., Hammond G. D. (1989). Improvement in survival after excision of primary tumor in stage III neuroblastoma.. J Pediatr Surg.

[OCR_01075] Hartmann O., Scopinaro M., Tournade M. F., Sarrazin D., Lemerle J. (1983). Neuroblastomes traités à l'Institut Gustave-Roussy de 1975 à 1979. Cent soixante-treize cas.. Arch Fr Pediatr.

[OCR_01080] Le Tourneau J. N., Bernard J. L., Hendren W. H., Carcassonne M. (1985). Evaluation of the role of surgery in 130 patients with neuroblastoma.. J Pediatr Surg.

[OCR_01090] Matthay K. K., Sather H. N., Seeger R. C., Haase G. M., Hammond G. D. (1989). Excellent outcome of stage II neuroblastoma is independent of residual disease and radiation therapy.. J Clin Oncol.

[OCR_01085] McGuire W. A., Simmons D., Grosfeld J. L., Baehner R. L. (1985). Stage II neuroblastoma--does adjuvant irradiation contribute to cure?. Med Pediatr Oncol.

[OCR_01095] Ninane J., Pritchard J., Morris Jones P. H., Mann J. R., Malpas J. S. (1982). Stage II neuroblastoma. Adverse prognostic significance of lymph node involvement.. Arch Dis Child.

[OCR_01100] Nitschke R., Smith E. I., Altshuler G., Altmiller D., Shuster J., Green A., Castleberry R., Hayes F. A., Golembe B., Ducos R. (1991). Postoperative treatment of nonmetastatic visible residual neuroblastoma: a Pediatric Oncology Group study.. J Clin Oncol.

[OCR_01107] Peto R., Pike M. C., Armitage P., Breslow N. E., Cox D. R., Howard S. V., Mantel N., McPherson K., Peto J., Smith P. G. (1977). Design and analysis of randomized clinical trials requiring prolonged observation of each patient. II. analysis and examples.. Br J Cancer.

[OCR_01111] Plantaz D., Rubie H., Michon J., Mechinaud F., Coze C., Chastagner P., Frappaz D., Gigaud M., Passagia J. G., Hartmann O. (1996). The treatment of neuroblastoma with intraspinal extension with chemotherapy followed by surgical removal of residual disease. A prospective study of 42 patients--results of the NBL 90 Study of the French Society of Pediatric Oncology.. Cancer.

[OCR_01119] Rosen E. M., Cassady J. R., Frantz C. N., Kretschmar C., Levey R., Sallan S. E. (1984). Neuroblastoma: the Joint Center for Radiation Therapy/Dana-Farber Cancer Institute/Children's Hospital experience.. J Clin Oncol.

[OCR_01134] Rubie H., Hartmann O., Michon J., Frappaz D., Coze C., Chastagner P., Baranzelli M. C., Plantaz D., Avet-Loiseau H., Bénard J. (1997). N-Myc gene amplification is a major prognostic factor in localized neuroblastoma: results of the French NBL 90 study. Neuroblastoma Study Group of the Société Francaise d'Oncologie Pédiatrique.. J Clin Oncol.

[OCR_01149] Tsuchida Y., Yokoyama J., Kaneko M., Uchino J., Iwafuchi M., Makino S., Matsuyama S., Takahashi H., Okabe I., Hashizume K. (1992). Therapeutic significance of surgery in advanced neuroblastoma: a report from the study group of Japan.. J Pediatr Surg.

[OCR_01156] West D. C., Shamberger R. C., Macklis R. M., Kozakewich H. P., Wayne A. S., Kreissman S. G., Korf B. R., Lavally B., Grier H. E. (1993). Stage III neuroblastoma over 1 year of age at diagnosis: improved survival with intensive multimodality therapy including multiple alkylating agents.. J Clin Oncol.

